# Development and validity of an educational folder for pulmonary tuberculosis sputum collection

**DOI:** 10.1590/0034-7167-2022-0194

**Published:** 2022-11-28

**Authors:** Karine Nascimento da Silva, Sabrina Alaide Amorim Alves, Maria do Socorro Vieira Lopes, Antonio Germane Alves Pinto, Maria Lúcia Duarte Pereira, Edilma Gomes Rocha Cavalcante

**Affiliations:** I Universidade Regional do Cariri. Crato, Ceará, Brazil; II Universidade Estadual do Ceará. Fortaleza, Ceará, Brazil

**Keywords:** Tuberculosis, Pulmonary, Educational Technology, Health Education, Validation Study, Sputum, Tuberculosis Pulmonar, Tecnología Educacional, Educación para la Salud, Estudio de Validación, Esputo, Tuberculose Pulmonar, Tecnologia Educacional, Educação em Saúde, Estudo de Validação, Escarro

## Abstract

**Objective:**

to develop and validate an educational folder for pulmonary tuberculosis sputum collection.

**Method:**

a methodological study, developed in four phases: project design, bibliographic survey, development, and content and appearance validity. For validity, the Content Validity Index greater than or equal to 0.80, the Kappa Coefficient and the Suitability Assessment of Materials were adopted.

**Results:**

an educational folder was developed containing the recommended steps for quality sputum collection. It was validated by 19 expert judges in the first validity cycle, obtaining an overall Content Validity Index of 0.90, perfect agreement among judges, with a total Kappa of 0.83 and superior suitability.

**Conclusion:**

the material is valid, as it contains suitable, simple and attractive language, illustrations and layout, indicating perfect agreement and reliability. Thus, it has the potential to assist in carrying out the recommended steps for correct sputum collection and enable the correct diagnosis.

## RESUMO

**Objetivo:**

desenvolver e validar um *folder* educativo para coleta de escarro da tuberculose pulmonar.

**Método:**

estudo metodológico, desenvolvido em quatro fases: elaboração do projeto, levantamento bibliográfico, desenvolvimento e validação de conteúdo e aparência. Para validação, adotaram-se o Índice de Validade de Conteúdo maior ou igual a 0,80, o Coeficiente Kappa e o *Suitability Assessment of Materials*.

**Resultados:**

desenvolveu-se um *folder* educativo contendo os passos recomendados para coleta de escarro de qualidade. Esse foi validado, por 19 juízes especialistas, no primeiro ciclo de validação, ao obter Índice de Validade de Conteúdo global de 0,90, concordância perfeita entre os juízes, com Kappa total de 0,83 e adequação superior.

**Conclusão:**

o material é válido, pois contém linguagem, ilustrações e *layout* adequados, simples e atraentes, indicando concordância e confiabilidade perfeita. Assim, apresenta potencialidade para auxiliar na realização dos passos recomendados para correta coleta de escarro e possibilitar o diagnóstico correto.

## RESUMEN

**Objetivo:**

desarrollar y validar una carpeta educativa para la recolección de esputo de tuberculosis pulmonar.

**Método:**

estudio metodológico, desarrollado en cuatro fases: diseño del proyecto, levantamiento bibliográfico, elaboración y validación de contenido y apariencia. Para la validación se adoptó el Índice de Validez de Contenido mayor o igual a 0,80, el Coeficiente Kappa y el Suitability Assessment of Materials.

**Resultados:**

se elaboró una carpeta educativa con los pasos recomendados para la recolección de esputo de calidad. Fue validado por 19 jueces expertos en el primer ciclo de validación, obteniendo un Índice de Validez de Contenido global de 0,90, perfecta concordancia entre los jueces, con un Kappa total de 0,83 y adecuación superior.

**Conclusión:**

el material es válido por contener lenguaje, ilustraciones y maquetación adecuados, sencillos y atractivos, indicando perfecta concordancia y fiabilidad. Por lo tanto, tiene el potencial de ayudar a llevar a cabo los pasos recomendados para la recolección correcta de esputo y permitir el diagnóstico correcto.

## INTRODUCTION

Tuberculosis (TB) is considered, in the world context, one of the main causes of death, with the pulmonary form being the most frequent and relevant in public health, as it maintains the disease transmission chain. Investigation for TB must be carried out through bacteriological tests which, with a positive result, confirms the active form of the disease, in addition to being fundamental in TB treatment management^(1)^.

The bacteriological tests used involve direct microscopic examination (direct smear microscopy), rapid molecular assay for tuberculosis (TRM-TB), culture for mycobacteria, identification and sensitivity test. In health services, the most used method is direct smear microscopy, as it is simple, safe and inexpensive to prove pulmonary TB diagnosis^(1)^. Furthermore, TRM-TB has advantages due to its easy handling, the potential to detect rifampicin resistance and a quick result^(2)^.

Thus, it is recommended that sputum collection, necessary for the assessment of these bacteriological exams, has an aspect, quantity and quality suitable for a correct assessment. The use of simple strategies that can be carried out in services daily increases the performance of these tests. In this context, the standardization of guidelines with their availability before sputum collection proves to be effective in increasing the performance of smear microscopy when compared to standard care^(3)^.

Health professionals’ practice, especially nurses, in providing guidelines for performing sputum collection, is capable of significantly increasing the bacteriological confirmation of the disease. Additionally, if implemented in a simple, gradual and effective way, it increases pulmonary TB diagnosis confirmation, even in the face of limited resources in Primary Health Care^(4)^. In this way, the strengthening of health education actions proves to be an effective strategy to reduce the delay in TB diagnosis^(5-6)^.

It is noteworthy that health education associated with the practical demonstration of sputum collection is necessary to improve quality. Thus, the use of educational technologies, culturally adapted to the needs of people with respiratory symptoms (RS) and with TB, makes it possible to improve knowledge for performing sputum collection, diagnosis, disease monitoring and subsequent treatment effectiveness^(7)^.

Thus, the use of technologies was identified in the scientific literature, such as videos^(8)^ and educational folders^(9)^, aiming to provide guidance on recommended steps for sputum collection. These demonstrated contributions to improve volume, quality and bacteriological confirmation. However, there is no evidence of educational technologies aimed at guiding all the steps necessary for sputum collection for people with RS and TB, developed and validated, considering the cultural context of Brazil.

In this sense, this study carried out the development and validity of an educational technology, of folder type, with information on the recommended steps for sputum collection in pulmonary TB. The technology has the potential to support the usual guidelines of health professionals on sputum collection at home.

## OBJECTIVE

To develop and validate an educational folder for pulmonary TB sputum collection.

## METHODS

### Ethical aspects

The study was approved by the Research Ethics Committee (REC), and participation in the research was legitimized by signing the Free and Informed Consent Term (ICF).

### Study design

This is a methodological research, developed from April 2019 to February 2021, carried out in four steps: 1) project elaboration and submission to the REC; 2) search in specialized literature; 3) elaboration of educational material;4) qualification with assessment of the material built by expert judges^(10)^. This survey was structured according to the Revised Standards for Quality Improvement Reporting Excellence (SQUIRE 2.0)^(11)^.

### Development steps

In the first step, study elaboration and submission to the REC was carried out. The second step refers to the investigation in specialized literature, through a survey of data in the materials prepared by the Ministry of Health, with the respective reading of manuals aimed at guidance for TB sputum collection^(1,12)^ and an integrative review.

It began with the identification of the Ministry of Health recommendations, as it is the institution that guides the guidelines that health professionals should provide to people with RS and TB in the sociocultural reality of Brazil. Thus, the guidelines to be recommended are standardized, but there is no correlation of each specific guideline with the importance of improving sputum appearance, quantity and quality. Then, the integrative review was carried out to identify the guidelines and the importance of carrying out all the recommended steps. It is noteworthy that the information obtained through the Ministry of Health and the integrative review were complementary and essential for educational folder development.

The integrative review was carried out by two researchers, independently, according to the Population, Variables and Outcomes (PVO) strategy, having as eligibility criteria for the selection of articles: people with RS and pulmonary TB aged from 18 years old (Population); guidelines performed (Variables); importance for improving the appearance, quantity and quality of sputum (Outcomes). With the strategy, the following guiding question was elaborated: what are the main guidelines and the importance of providing them to people with RS and pulmonary TB for quality sputum collection?

The inclusion criteria established were: qualitative or quantitative articles that met the eligibility criteria of the search strategy. Theses, dissertations, response letters and editorials were excluded, as it was considered that the original articles resulting from the search would properly answer the guiding question. There were no restrictions on language or year of publication of analyzed studies.

The search was carried out in a paired manner, from October to November 2019, through the Coordination for the Improvement of Higher Education Personnel (CAPES) journals portal, in the Medical Literature Analyzes and Retrieval System Online (MEDLINE), Latin American Literature in Health Sciences (LILACS), National Library of Medicine National Institutes of Health (PubMed), Scopus and Web of Science (WoS) databases, using the advanced search method and categorizing title, abstract and subject. For the search, MeSH descriptors were used in PubMed.

The following search strategies were used to expand the scope of studies that could answer the guiding question of this research, according to each database: in PubMed, the search strategy used corresponded to 1) (“Respiratory Symptomatic”) AND “Health Education”; 2) (“Respiratory Symptomatic”) AND Sputum; 3) (“Health Education”) AND Sputum; 4) (“Tuberculosis, pulmonary”) OR ((“Respiratory Symptomatic”)) AND “Health Education” AND Sputum.

In the third step, the educational material elaboration was carried out, in which the content, illustrations and diagramming were developed, based on aspects of language, illustration and layout^(13)^. The vocabulary used was inviting, easy to understand and coherent with the message to be conveyed to the target audience^(13-14)^. For the choice of illustrations, dynamic, attractive images with good quality and color were sought. For illustration elaboration and diagramming, Adobe Photoshop and Adobe Illustrator were used by two graphic designers.

The fourth step, material qualification, was carried out through the validity of content and appearance regarding the criteria of clarity, theoretical relevance and practical relevance^(15)^, in addition to assessing the educational folder suitability and difficulty through the Suitability Assessment of Materials (SAM) by expert judges. Judge selection took place, according to Jasper recommendations^(16)^, by inviting health professionals from different areas of knowledge, considering their experience and qualifications in public health, pulmonary TB, sputum collection and the development of educational materials.

The number of expert judges was defined by Pasquali’s recommendations^(15)^, which suggests six to twenty people. With regard to expert identification and selection, this began with a search using the *Curriculum Lattes* system, using the professional action filter strategy, selecting: major area: health sciences; area: nursing; subarea: nursing in emerging, reemerging and neglected diseases; subject: pulmonary TB; at the bases: doctors and other researchers; Brazilian nationality. Also, snowball or convenience sampling was used as a strategy for judge selection^(17)^.

After being selected, 71 people eligible for judges received an invitation letter, sent via email, with information about the research and its objectives. Upon confirmation of participation of 19 judges, a kit containing the educational folder prepared, the two-way informed consent form and a link to access the validity protocol in Google Docs, with a questionnaire to characterize the judges, content validity and appearance and assessment through SAM.

The validity instrument contained the criteria of language clarity, theoretical relevance and practical relevance. In terms of language clarity, judges were asked to assess the language used in each step, considering the educational folder target population characteristics. With regard to theoretical relevance, the degree of association of illustrations, texts and the theoretical aspect of pulmonary TB sputum collection were considered. As for practical relevance, assessment of illustrations and texts and their practical importance for carrying out sputum collection was requested^(15)^.

To make the material qualification process more appropriate, it was decided to carry out assessment according to each item and orientation. For each item assessed, the judges could classify it: 1) totally disagree; 2) disagree; 3) neither agree nor disagree; 4) agree; 5) totally agree. At that moment, the judges could make suggestions and propose changes in an appropriate space for this purpose. In addition, for each item with a “disagree” or “strongly disagree” response, suggestions for changes were requested to improve the items. Also, in the fourth part of the assessment instrument, items were assessed regarding content, language, illustration, layout and presentation, learning encouragement and cultural suitability.

The expert judges had 7 days to return the material. Those who do not respond to the validity protocol within the stipulated period received e-mails periodically, reminding them sending it and the importance of carrying out the assessment within the defined deadline.

### Data analysis

In the content validity data analysis with expert judges, the Content Validity Index (CVI) was used^(17)^. Items that reached a CVI greater than or equal to 0.80 were considered validated in terms of clarity of language, practical relevance and theoretical relevance and general assessment of the educational folder. Also, items that received suggestions for alteration or score 1, 2 or 3 were reviewed and changed, according to suggestions and search in the pertinent literature.

Furthermore, analysis was performed using the Kappa coefficient to assess the educational folder reliability by inter-rater agreement, assessing the agreement among judges’ answers. The Kappa coefficient was calculated by the ratio of the proportion of times judges agreed to the maximum proportion of times they could agree. Thus, the Kappa coefficient agreement determination followed the following recommendation: < 0, there is no agreement; 0 - 0.20, minimum agreement; 0.21 - 0.40, reasonable agreement; 0.41 - 0.60, moderate agreement; 0.60 - 0.80, substantial agreement; and 0.81 - 1.0, perfect agreement^(18)^. The level of significance was set at 5% (p<0.05).

The data obtained through the SAM were composed of six categories: content; language/literacy requirement; illustrations; layout and presentation; learning encouragement/motivation; and cultural suitability. They were assessed as superior, with two points, suitable, with one point, and unsuitable, with no point. Thus, the educational folder was considered superior, with a score between 70% and 100%, suitable, with a score between 40%-69%, and unsuitable, with a score between 0-39%^(14)^.

After validity with the expert judges, the analysis of the suggestions made in each item and adjustments to the material was carried out, thus resulting in a new version of the educational folder.

## RESULTS

### Specialized literature search

Of the 1,536 studies articles identified in the electronic databases, 55 were selected for full reading, 44 in PubMed, 10 in Scopus and one in the Web of Science. After this phase, 11 included studies were selected for final analysis. Eleven studies were analyzed, which made it possible to identify the recommended guidelines, the influence of educational interventions, the difficulties and suggestions of strategies to improve sputum sample quality, quantity and aspect. It is noteworthy that, for the development of the educational folder, it was considered that there are ideal and specific steps to carry out sputum collection, which were listed through literature search.

The search for evidence from the integrative review and reading of the Ministry of Health manuals made it possible to identify the main guidelines that should make up the educational folder, based on the three moments for sputum collection: a) general guidelines (before sputum collection); b) guidelines for expectoration (time of sputum collection); c) guidelines for after collection (delivery of material to the Health Unit) and the importance of providing them to obtain a sample with the recommended aspect, quantity and quality. Moreover, it was possible to identify the main perceived difficulties, suggestions for quality sputum collection strategies and the aid of educational technologies.

### Educational folder preparation

The educational folder was prepared in two sheets of A4 size (21cm x 29.7cm), divided into three parts, so that it could be folded when printed, totaling 6 pages, with landscape orientation. Font Times New Roman, size 12, was used in the body of the text, 14 in the topics and 20 in the title. The title “Sputum examination for pulmonary tuberculosis investigation: home collection guidelines” addressed the main purpose of this educational folder.

**Figure 1:**
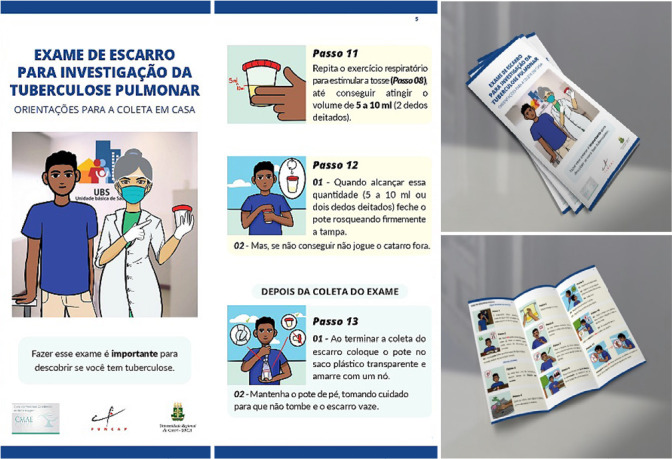
Representative illustrations and printed simulation of “Sputum examination for pulmonary tuberculosis investigation: home collection guidelines”, Crato, Ceará, Brazil, 2021

On each page, up to four main logical sequence ideas were presented to the reader, containing the recommended steps for sputum collection. The main information was made available with an objective demonstration of the expected action, avoiding the accumulation of guidelines. The sentences were structured in a way that simulated a conversation, with short, simple sentences and use of active voice. The ideas were exemplified through illustrations, which simulated the performance of each step presented in the folder, being organized close to the written guidelines, according to Figure 1.

### Qualification with material assessment by expert judges

In the validity step, 19 judges participated, all female, aged between 28 and 52 years and mean age of 38.7 years (SD ± 9.2). As for the region of operation, 10 (52.5%) were from the Northeast, most of them from the state of Pernambuco. With regard to the undergraduate course, all (100.0%) were graduated in nursing, with 2 (10.6%) of these graduates also from another higher education course. Regarding the degree, 10 (52.6%) had at least a doctoral degree.

All judges have experience in teaching, assisting or managing TB and/or sputum collection and educational technologies. Thus, 16 judges (84.2%) develop or have developed studies on TB and/or sputum collection, and 14 (73.7%) develop or have developed studies on educational intervention or educational technologies. Also, 18 (94.7%) work in the care of people with TB and guide people with RS on the steps for sputum collection, and 17 (89.4%) participate in research groups on TB, sputum collection, educational intervention or educational technologies.

The educational folder obtained CVI-I of 0.87 for language clarity, 0.92 for theoretical relevance and 0.92 for practical relevance. Thus, an overall CVI of 0.90 was obtained, demonstrating the excellent level of agreement among judges. Thus, the judges validated the first version of the educational folder, as shown in Table 1.

**Table 1:** Distribution of each item’s Content Validity Index, Crato, Ceará, Brazil, 2021

Educational folder items	Language clarity	Theoretical relevance	Practical relevance
Cover: S-CVI/UA^*^	0.79	0.79	0.84
Item 1: S-CVI/UA^*^	0.79	1	0.95
Item 2: S-CVI/UA^*^	0.58	0.89	0.79
Item 3: S-CVI/UA^*^	0.84	0.84	0.84
Item 4: S-CVI/UA^*^	1	0.95	0.95
Item 5: S-CVI/UA^*^	0.89	0.89	0.84
Item 6: S-CVI/UA^*^	0.89	0.89	0.95
Item 7: S-CVI/UA^*^	0.84	0.89	0.95
Item 8: S-CVI/UA^*^	0.95	0.95	0.95
Item 9: S-CVI/UA^*^	0.89	0.95	0.95
Item 10: S-CVI/UA^*^	0.89	0.95	0.89
Item 11: S-CVI/UA^*^	1	1	1
Item 12: S-CVI/UA^*^	0.79	0.89	0.84
Item 13: S-CVI/UA^*^	0.89	1	1
Item 14: S-CVI/UA^*^	0.95	0.89	0.95
Item 15: S-CVI/UA^*^	0.89	1	1
Item 16: S-CVI/UA^*^	0.89	0.89	0.95
Folder as a whole:			
S-CVI/UA^*^	0.89	0.89	1
I- CVI^**^	0.87	0.92	0.92
S-CVI/Ave^***^	0.90		

* S-CVI/UA - proportion of scale items that reached scores 4 - agree and 5 - totally agree;

** I-CVI - content validity of individual items;

*** S-CVI/Ave - average of validity indices for all scale indices.

Then, we proceeded to assessment, according to the Kappa coefficient, to estimate the agreement among judges’ answers and assess the educational folder reliability. The total Kappa greater than 0.81 was evidenced, indicating an excellent agreement and reliability, according to Table 2.

**Table 2:** Kappa Coefficient distribution. Crato, Ceará, Brazil, 2021

Items	Language clarity	Theoretical relevance	Practical relevance	Kappa Coefficient	95% CI	*p* value
Cover	0.65	0.65	0.72	0.67	0.57 - 0.77	0.001
Item 1	0.65	1.0	0.90	0.85	0.40 - 1.00	0.015
Item 2	0.48	0.80	0.65	0.64	0.25 - 1.00	0.020
Item 3	0.72	0.72	0.72	0.72	^*^	^*^
Item 4	1.00	0.89	0.89	0.93	0.78 - 1.00	0.001
Item 5	0.80	0.80	0.72	0.77	0.65 - 0.89	0.001
Item 6	0.80	0.80	0.89	0.83	0.70 - 0.96	0.001
Item 7	0.80	0.89	0.89	0.86	0.73 - 0.99	0.001
Item 8	0.89	0.89	0.89	0.89	^*^	^*^
Item 9	0.80	0.89	0.89	0.86	0.73 - 0.99	0.001
Item 10	0.80	0.89	0.80	0.83	0.70 - 0.97	0.001
Item 11	1.00	1.00	1.00	1.00	^*^	^*^
Item 12	0.65	0.80	0.71	0.72	0.53 - 0.91	0.004
Item 13	0.80	1.00	1.00	0.93	0.65 - 1.00	0.005
Item 14	0.89	0.80	0.89	0.86	0.73 - 1.00	0.001
Item 15	0.80	1.00	1.00	0.93	0.65 - 1.00	0.005
Item 16	0.80	0.89	0.89	0.83	0.70 - 0.97	0.001
Whole	0.80	0.80	1.00	0.87	0.58 - 1.00	0.006
Total Kappa^^**^^	0.80	0.86	0.86	0.83		

* Values did not vary;

** Total Kappa performed using average of individual values; 95% CI - Confidence Interval.

Fifty suggestions for modifications were made, of which 12 turned to illustrations and 38 to the educational folder content. Among these, the main ones refer to clarity improvement, with suggestions for replacement by simpler and easier to understand terms, in most items. Moreover, it was suggested to add information that explained the reason for carrying out the guided steps and changes in the illustrations of seven of the 18 items that make up the material.

The suggestions were analyzed according to the integrative review, guidelines from the Ministry of Health and a pertinent search in the scientific literature, in search of updates on the subject. Thus, 35 suggestions were accepted, and 17 were not accepted, according to Chart 1.

**Chart 1:** Suggestions for changes made by expert judges, Crato, Ceará, Brazil, 2021

Item	Changes suggested by expert judges	Assessment
Cover	Replace “domicile” with a word that is easier to understand (J2).	Accepted
	Replace the title for “Sputum examination for pulmonary tuberculosis investigation: home collection guidelines” (J4).	Accepted
	In the subtitle: Replace with “to find out if you have tuberculosis” (J5).	Accepted
	In the illustration: arrange the character’s hair, less color in the mask and highlight the collection pot, with the lowest hand preferably (J4).	Accepted
	Add the user something that portrays the home environment (J2).	Accepted
	Remove the mask from the professional (J5).	Not accepted
	At the bottom of the cover, place a watermark with the drawings of Koch’s bacilli (J8).	Not accepted
1 Fluid intake	Complement with: “the more water to drink, the better” (J3).	Not accepted
	It is important to drink plenty of fluid (water, tea, juice) (J4).	Not accepted
	Explain the reason for drinking too much water (J5).	Accepted
	Replace with “greater volume of water” (J11, J15).	Accepted
2 Food and fluid intake	I suggest that in the illustration the cup and the plate are empty, to facilitate the understanding of fasting (J2).	Not accepted
	In the image, add a glass of water with a green light (allowed) (J3, J15).	Not accepted
	Review the term “sample” (J3, J10, J11).	Accepted
	I suggest changing “sample” by “mixed with phlegm at the time of collection” (J3).	Accepted
	Replace the term “eat 6 hours before” with “eat before” (J4, J5).	Accepted
	Add the term “preferably” (J11).	Accepted
	Mention that water intake should be carried out the night before and not on the day of collection (J18).	Not accepted
3 Medication intake	Replace the tablets with a sputum collection jar (J4).	Not accepted
	Add the term “of exam collection” (J15).	Accepted
	Make it clearer in the image the “cannot” (J15).	Accepted
4 Hand hygiene	I suggest removing the comma before soap and water in the sentence (J2, J3).	Accepted
	Add “examination collection” (J3, J15).	Accepted
	Add “on clean towel or cloth” (J18).	Accepted
6 Hand hygiene	I suggest putting the word “dentures” in parentheses (J4).	Accepted
	Not removing the dental prosthesis does not change the exam (J5).	Not accepted
7 Oral hygiene	Add a tap with water (J2).	Accepted
	Place oral hygiene only with mouthwash with water (J4, J18).	Accepted
	I suggest explaining that the person cannot use toothpaste because it can change the exam (J5).	Accepted
8 Site	Add the words “illuminated and ventilated” (J3).	Accepted
	No need to look for an open and reserved place (J5).	Not accepted
9 Technique	Add “pull the air” (J3).	Accepted
	Add “01. Hold the collecting pot in hand with the lid open” (J4 and J5).	Accepted
10 Technique	Replace with “make the effort to cough, directing the sputum into the pot” (J4 and J5).	Accepted
	Replace the sputum illustration (J5).	Accepted
11 Technique	No suggestions.	-
12 Volume	Add “if necessary” (J3).	Not accepted
	Replace “steps” with “breathing exercise” (J4).	Accepted
	Replace with “2 fingers of pot height”, adding the mark of a person measuring with 2 fingers lying down (J4, J5).	Accepted
	Add 5 ml to the drawing (J10, J15).	Accepted
13 Volume	Add: but even if you can’t, don’t throw away the phlegm (J3).	Accepted
	Add “5-10 ml or two fingers lying down” (J4).	Accepted
14 Packaging	Replace “after” with “to” (J2).	Accepted
	Replace “examination” with “sputum collection” (J4).	Accepted
15 Packaging	Replace “opaque” with “dark” (J3, J4, J10).	Accepted
	Illustrate the Health Unit on the way (J10).	Accepted
16 Delivery	In the illustration, take into account biosafety standards (J4).	Accepted
	Add figure 15 and 16 or add “2h” to figure 16 (J10).	Accepted
	Indicate where the refrigerator should be (J5).	Accepted
Whole	Add “the best sample is the one performed in the morning, upon waking up” (J5).	Accepted

In assessing the educational folder suitability, the content criteria (SAM=84%), language (SAM=72%), illustration (SAM=85%), presentation (SAM=73%), learning encouragement (SAM=75 %) and cultural suitability (SAM=76%) were considered superior, as they obtained a percentage higher than 70%. Furthermore, the overall educational folder assessment (SAM=77%), obtained through the average of the individual criteria, demonstrates that the material as a whole was considered superior, according to Table 3.

**Table 3:** Suitability Assessment of Materials scores, Crato, Ceará, Brazil, 2021

Domains	2 scores (superior)	1 score (suitable)	0 score (unsuitable)	NA^*^	Total (%)
1. Content					84
a) Purpose is evident	15	4	-	-	89
b) Content addresses behaviors	8	10	-	1	72
c) Content focus on purpose	15	3	1	-	87
d) Content highlights key points	15	3	1	-	87
2. Suitable language					72
a) Reading level	5	13	1		61
b) Writing in active voice	8	11			71
c) Common vocabulary	7	12			68
d) Context comes before new information	8	9	1	1	69
e) Learning facilitated by topics	15	4	-		90
3. Graphic illustrations					85
a) Purpose is clear	14	5	-		87
b) Types of illustrations	13	5	1		82
c) Illustrations are relevant	14	5	-		87
4. Layout and typography					73
a) Layout characteristics	11	8	-		79
b) Font size and type	11	8	-		71
c) Subtitles are used	7	12	-		68
5. Learning encouragement and motivation					75
a) Uses interaction	7	12	-		68
b) Guidelines are specific and give examples	12	7	-		82
c) Motivation and self-efficacy	10	9	-		76
6. Cultural suitability			-		76
a) Is similar to its logic, language and experience	11	7	1		76
b) Cultural image and examples	10	9	-		76

* *NA - not applicable.*

## DISCUSSION

The educational folder “Sputum examination for pulmonary tuberculosis investigation: home collection guidelines”, developed after a specialized search in the scientific literature, was validated in the first validity cycle by a significant number of judges, with these 19 experts having extensive theoretical and practical experience on pulmonary TB, sputum collection and educational technology.

Through scientific literature search, it was established that there are ideal steps to be taken to obtain quality sputum and correct diagnosis of pulmonary TB. Testing conduction depends on a quality sputum sample, which is influenced by the way the steps are performed. Proper instruction on how to produce a sputum sample substantially increases TB diagnosis, resembling innovative and sophisticated laboratory tests, which justifies the incorporation of these interventions in services’ daily routines^(4)^.

When considering continuing instructions on sputum collection at the time of the second sputum sample for RS or control persons during TB treatment, which are carried out especially in the domestic environment, with the practical importance of educational technologies, the educational folder was prepared. Thus, this technology is a driving tool for health promotion practices, allowing access to the information necessary for autonomy and co-responsibility of people with RS or those undergoing TB treatment in self-care actions.

Thus, the educational folder was developed in a logical sequence, with short sentences, active voice and illustrations, to demonstrate the recommended steps for sputum collection. To this end, a systematized and organized construction process was followed, to give greater reliability to contain the essential information for understanding the target audience. In addition to this, the culture and socioeconomic reality of the educational material target audience were considered, thus avoiding abstract words, complex sentences and passive voice use.

The use of CVI parameter higher than 0.80 was evidenced to define the validity criterion of several educational technologies^(19-20)^. The folder was considered validated for content and appearance, constituting an important tool to assist in educational processes and health care.

In the present study, an excellent educational folder agreement and reliability was identified in assessment, according to the total Kappa Coefficient, being greater than 0.81. Among the measures used in the validity process, the Kappa reliability index was verified as a measure increasingly used in the production and dissemination of scientific knowledge in nursing^(21-22)^.

Regarding the suggestions made by the judges, recommendations were made to improve item clarity in terms of replacing them with simpler and more easily understood terms in most items in the educational folder. All suggestions made were accepted in order to facilitate the understanding of information provided. Unaccepted changes refer to those that repeated the information mentioned above, which generates accumulation in the material, which can make it tiring and redundant.

It was suggested to add information that would explain the reason for performing the guided steps. It is noteworthy that access to this information is essential to guarantee TB diagnosis, treatment and management in the most susceptible communities, in which the distribution of educational technologies becomes a powerful and low-cost tool to guarantee access^(23)^. The grammatical alteration of an item was recommended when considering the recommendation for proofreading to improve validity indices^(24)^.

Changes were also suggested in the illustrations of seven of the 18 items that make up the material. Among these, all those that facilitated the performance of the recommended step were accepted, while those in which there was no recommendation in the literature were not accepted. The use of educational technologies that provide the necessary steps to acquire a quality sputum sample, such as educational videos, helps to improve the understanding of why sputum collection is performed, encouraging achievement as directed, which leads to improvements in TB diagnosis, monitoring and treatment effectiveness^(23)^.

Thus, the main changes perceived in the educational folder, according to the judges’ suggestions, were the cover, items 1 (orientation for water intake) and 2 (food), which received a CVI of less than 0.80 regarding the clarity of language. The changes were accepted considering the relevant scientific literature on the subject and to facilitate the understanding of the target audience of educational technology, seeking to make it understandable for people with different educational levels.

In the educational folder suitability overall assessment through the SAM, the material was considered superior, as it obtained an assessment of more than 70%. It is noteworthy that, in the individual assessment regarding the criteria of content, language, illustration, presentation, learning encouragement and cultural suitability, they were also assessed as superior. Furthermore, changes were made to improve domain qualification and the understanding of the people who will use the educational material.

In assessment through the SAM, a study that developed an educational booklet to promote self-efficacy in Zika prevention corroborates the judges’ analysis with the classification of educational technology, mostly as superior, obtaining the SAM with an average of 75%^(24)^.

Thus, the educational folder “Sputum examination for pulmonary tuberculosis investigation: home collection guidelines” is a validated educational technology, as it contains suitable, simple and attractive language, illustrations and layout for people who will perform sputum collection for pulmonary TB diagnosis. Thus, it is expected that the educational folder will be used in care practice, being provided by professionals and health services in conjunction with oral instructions, to facilitate the realization, in a home environment, through access to printed guidelines, with accessible language and illustrations that simulate the performance of each recommended step.

### Study limitations

The subjective process of content validity is pointed out as a limitation, with emphasis on the need for validity with the target audience. However, the result does not preclude the educational folder development and content validity, being steps prior to validity with the target audience. Thus, we indicate, for subsequent studies, the translation of content to an interactive digital platform and validity with the target audience.

### Contributions to nursing, health, and public policies

We believe that this study presents as contributions the detailing of the development process and the validity of a printed educational technology, helping to produce, with scientific rigor, other technologies for nursing care, strengthening it as the science of care. Moreover, it is noteworthy that developing and validating the educational folder made it possible to develop a technology with suitable, simple and attractive language, illustrations and layout. Thus, it provides the necessary information to perform the recommended steps for sputum collection.

## CONCLUSIONS

The development of educational technology was carried out with scientific rigor, based on the most appropriate evidence for the research object. The educational folder “Sputum examination for pulmonary tuberculosis investigation: home collection guidelines” had its content and appearance considered relevant and validated in terms of clarity of language, with theoretical relevance and practical relevance evidenced by the overall CVI of 0.90. Furthermore, the agreement among judges’ answers in the educational folder obtained perfect agreement and superior suitability.

Thus, our aim is that this folder be used in care practice, being provided by professionals and health services in conjunction with oral instructions, facilitating access to the information necessary for acquisition of a sputum sample with volume, quantity and aspects recommended for bacteriological confirmation of the disease.

## SUPPLEMENTARY MATERIAL

Developed as a section of a dissertation entitled “*Construção e validação de um folder educativo para coleta de escarro da tuberculose pulmonar*” of an academic master’s degree in nursing at the *Universidade Regional do Cariri*.
